# Sexual dimorphism and sex-biased gene expression in an egg parasitoid species, *Anastatus disparis*

**DOI:** 10.1186/s12864-020-06903-5

**Published:** 2020-07-18

**Authors:** Peng-Cheng Liu, De-Jun Hao, Hao-Yuan Hu, Jian-Rong Wei

**Affiliations:** 1 The College of Ecology and Environment, Anhui Normal University, Wuhu, Anhui Province China; 2 The College of Forestry, Nanjing Forestry University, Nanjing, Jiangsu Province China; 3 The College of Life Science, Hebei University, Baoding, Hebei Province China

**Keywords:** Transcriptomic analysis, Sex-specific genes, Flyability, Longevity, Aggression, Venom protein, Biological control

## Abstract

**Background:**

Differences in the expression of genes present in both sexes are assumed to contribute to sex differences including behavioural, physiological and morphological dimorphisms. For enriching our knowledge of gender differences in an important egg parasitoid wasp, *Anastatus disparis* (Hymenoptera: Eupelmidae), sex-biased differences in gene expression were investigated using Illumina-based transcriptomic analysis.

**Results:**

A total of 15,812 resulting unigenes were annotated, and a large set of genes accounting for 50.09% of the total showed sex-biased expression and included 630 sex-specific genes. Gene Ontology (GO) enrichment analyses showed that the functional categories associated with sex-biased genes were mainly related to reproduction. In addition, the transcriptome data provided evidence that sex pheromones in *A. disparis* are produced by the female, and activity of Δ12-desaturases appear to have been replaced by Δ9-desaturases playing roles in sex pheromone production. The large set of sex-biased genes identified in this study provide a molecular background for sexually dimorphic traits such as flyability, longevity, and aggression in this species and suggests candidate venom proteins expressed only in females that could be used for biological control.

**Conclusions:**

This study provides comprehensive insight into sexually dimorphic traits of a parasitoid wasp and can inform future research into the molecular mechanisms underlying such traits and the application of parasitoids to the biological control of pest species.

## Background

Sex differences, including behavioural [1], physiological [2], and morphological dimorphisms [3], occur in a broad range of animal and plant species. It is often assumed that most of these phenotypic differences are mediated by differences in the expression of genes present in both sexes [4, 5]. Genes that are expressed in both sexes but at a higher level in one sex have been termed sex-biased genes, which can be further separated into male-biased and female-biased genes, depending on which sex shows higher expression [6]. This differential gene expression may involve a significant proportion of the genome [7–10]. For example, when whole adult females and males of *Drosophila melanogaster* are compared, up to 57% of their genes show sex-biased expression [8]. In addition, research in *Drosophila* reveals that the genes expressed differentially tend to be male biased [11]. However, the finding of more male-biased genes in *Drosophila* may be confounded by the inclusion of sex-specific genes [12]. In contrast, studies in *Tribolium castaneum* and *Anopheles gambiae* [13, 14] have shown that many of the sex-biased genes in these species are female biased. In general, male-biased genes evolve more rapidly than female-biased genes [15–18], suggesting that males experience stronger positive selection than females [4, 5], although the genetic architecture of gene expression and other factors might also explain the more rapid evolution of male-biased genes [19]. Sex-biased gene expression has been documented in a range of different species, including brown algae [20], birds [21, 22], nematodes [23], *Daphnia pulex* [10], cichlid fishes [24], guppies [25], moths [26], the pea aphid [27], and multiple insect species [8, 28–33]. Studies such as those listed above provide a comprehensive overview of sex-biased gene expression in a broad range of species and offer more useful information to determine sexual dimorphism.

Haplodiploid parasitoid species are important insects that parasitize other organisms [34] and have been extensively applied to reduce the population sizes of pest species [35–40]. However, sex differences in gene expression have not been investigated in parasitoid species other than the model organisms *Nasonia* species [41]. Sex-biased genes differ between non-haplodiploid and haplodiploid. In most species, the male and female genomes differ in the genes located on sex-specific chromosomes (such as the Y chromosome of mammals) [4, 5]. However, sex determination in hymenopteran species is haplodiploid: males develop from unfertilized eggs and are haploid, whereas females develop from fertilized eggs and are diploid [42]. Therefore, in haplodiploid species, females and males are nearly identical genetically [6]. As a result, most differences between females and males of haplodiploid species are due to differences in gene dosage. Furthermore, sex-biased genes in haplodiploid species, e.g., *Nasonia* spp., involve a significant proportion of the genome and a greater proportion than observed in species with sex chromosomes [41].

*Anastatus disparis* (Hymenoptera: Eupelmidae) is an egg parasitoid species, which can parasitize the eggs of several noxious species of Lepidoptera, including *Lymantria dispar*, *Antheraea pernyi*, *Odonestis pruni*, and *Actias selene ningpoana* [43, 44]. It has been considered a potential biological control agent of the gypsy moth in North America [43, 45]. Sexual dimorphisms related to behaviour, physiology, and morphology in *A. disparis* have been extensively described. First, although wasp body size is correlated with host quality [44, 46], the body size of females is typically significantly larger than males. Females can live more than 1 month in the field and lay hundreds of eggs over their lifetime, while males live for only approximately 5–7 days and exhibit frequent and extreme fighting behaviour to acquire mating opportunities [43, 46, 47]. Third, females do not fly but jump very quickly, while males can fly short distances [45, 47]. In the present study, the sexually dimorphic traits of this parasitoid wasp (e.g., longevity, flyability) were investigated at the transcriptome level to improve our understanding of their biological traits. The transcriptome data revealed a series of candidate genes that can aid future studies of biological traits in *A. disparis*. Importantly, males of few parasitoid wasps other than *A. disparis* exhibit extreme fighting behaviour [48, 49]. Thus, we expected that transcriptome data could provide a molecular background and knowledge on the male fighting behavior, and potential candidate genes for future studies of the molecular and evolutionary mechanisms underlying extreme fighting. As a parasitoid species, *A. disparis* has been applied as a biological control agent [44–47]. This study also attempted to explore the potential application, e.g., venom proteins identified from the transcriptome data for improving biological control of pest species.

## Results

### Sex-biased genes

Using our assembled transcriptome as a reference, we identified putative genes expressed in males and females using the Fragments Per Kilobase of transcript per Million mapped reads (FPKM) method. Genes with FPKM> 1 in at least one sample and a false discovery rate (FDR) < 0.05 were defined as significant sex-biased (SB) genes [41]. By comparing the female and male transcriptomes, 7920 SB genes in *A. disparis* were found, accounting for 50.09% of the total (7920/15812) (Table 1). Previous studies in *T. castaneum* and *A. gambiae* [13, 14] showed that many annotated sex-biased genes were female-biased. In contrast, in our study and consistent with results in *Nasonia* species, the numbers of female- and male-biased genes were approximately equal, being 4310 (**Table S****1**) and 3610 (**Table S****2**, respectively).

**Table 1:** Summary of number of annotating genes in sex-biased categories

Sex-biased expression category	Criteria	Number	Proportion (%)
Annotated genes in RNA-seq	FPKM> 1 at least one replicate	15,812	–
Female-biased genes	*q* < 0.05; female-biased	4310	27.258%
Male-biased genes	*q* < 0.05; male-biased	3610	22.831%
Female-specific genes	FPKM< 0.4 in males; FPKM> 2 in females	518	3.276%
Male-specific genes	FPKM< 0.4 in females; FPKM> 2 in males	112	0.708%

**Fig. 1:**
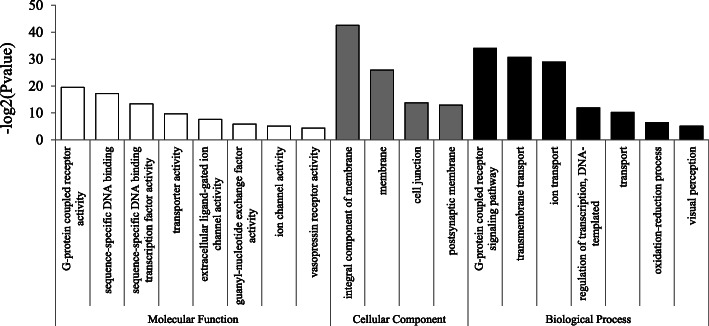
GO enrichment analysis of male-biased genes. GOSeq was used for the GO enrichment analysis, and an adjusted Q-value < 0.05 was chosen as the significance cutoff

Gene Ontology (GO) analysis suggested that the identified SB genes were highly enriched for GO terms associated with reproductive functions. Nineteen subcategories were enriched in male-biased genes (Fig. 1**)**, which may be mainly related to sperm. For example, the enrichment of these genes in subcategories involving the encoding of transport proteins (i.e., transmembrane transport; transporter activity) might be consistent with the motile nature of sperm [11]. The high over-representation of gene products associated with membranes (integral component of membrane; membrane) is likely due to the requirements of the sperm axoneme structure [11]. In addition, the enriched subcategory of “oxidation-reduction process” might also provide supporting evidence, which in males are needed to provide an environment supportive of sperm viability and motility and to counter the oxidative effects of sperm catabolism [50, 51].

**Fig. 2:**
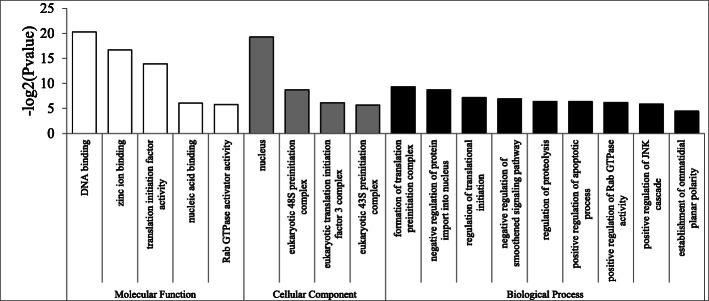
GO enrichment analysis of female-biased genes. GOSeq was used for the GO enrichment analysis, and an adjusted Q-value < 0.05 was chosen as the significance cutoff

Eighteen subcategories were enriched in female-biased genes, as shown in Fig. 2. Consistent with previous studies in *Drosophila*, mosquitoes and *Daphnia* [8–10], many subcategories of the biological process (BP) category were assigned to genes encoding proteins involved in ribosomal function, translation initiation, and DNA replication, which might be related to egg production in females [11, 52, 53]. Genes encoding translational initiation complexes and ribosomal content were highly expressed in females and might associated with maternal loading into the egg. Indeed, mutations in many ribosomal protein genes result in female sterility due to defective egg formation [53]. The over-representation of transcripts from genes required for DNA replication may be required for nurse cell polyploidization or the rapid division of embryonic cells, which rely on maternally deposited gene products [11, 52].

### Sex differences in flyability, longevity and aggression

Several other of the identified SB genes provide insight into sexually dimorphic traits of *A. disparis* and include candidate genes that can be studied in the future; these are described below.

**Fig. 3:**
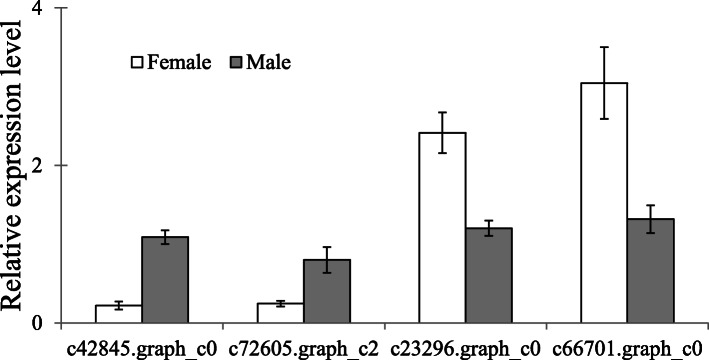
Sex differences in expression of the annotated genes *vestigial*, *nubbin*, *trehalase* and *seryl-tRNA synthetase*, which are involved in flyability, as determined by qRT-PCR. The expression of genes determined through qRT-PCR was calculated by the 2^-ΔΔCt^ method using the housekeeping gene EF1A as a reference to eliminate sample-to-sample variation in the cDNA samples

Transcriptome data suggest a molecular background for why the two sexes of this species have distinct flight performance with males can fly short distances, while females do not fly [43, 45, 47]. Result of quantitative real time polymerase chain reaction (qRT-PCR) showed that the protein-coding genes *vestigial* (Fig. 3**,** c42845.graph_c0; qRT-PCR: *t* = − 4.799, *df* = 4, *p* < 0.05) and *nubbin* (Fig. 3**,** c72605.graph_c2; qRT-PCR: *t* = − 4.507, *df* = 4, *p* < 0.05) highly expressed in *A. disparis* males; both have been identified as important for the physical development of wings in *Drosophila* [54]. Furthermore, studies of aphids have revealed that *trehalase* and *seryl-tRNA synthetase*, which are involved in the conversion of trehalose to glucose [55] and tRNA metabolic processes [56], respectively, are related to flightless morphs. In the present study, the transcriptome data showed that genes encoding *trehalase* (c23296.graph_c0) and *seryl-tRNA synthetase* (c66701.graph_c0) were highly expressed in (flightless) females; these results were verified by qRT-PCR (Fig. 3: c23296.graph_c0, *t* = − 3.169, *df* = 4, *p* < 0.05; c66701.graph_c0, *t* = 3.396, *df* = 5, *p* < 0.05).

**Fig. 4:**
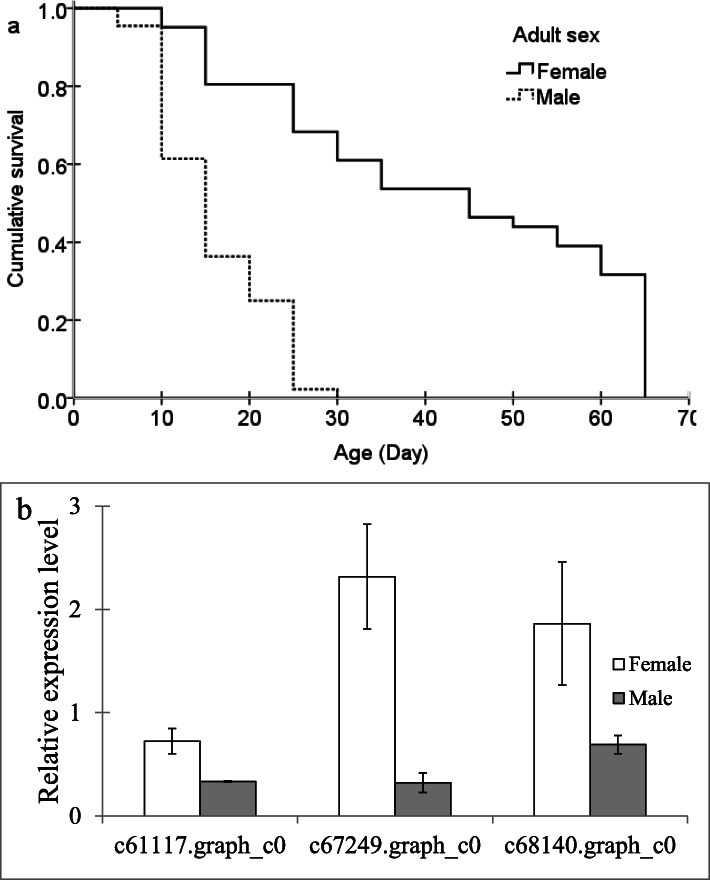
Sex differences in longevity (**a**) and expression as determined from qRT-PCR of annotated potential genes encoding superoxide dismutase and *vitellogenin* (**b**). The expression of genes determined through qRT-PCR was calculated by the 2^-ΔΔCt^ method using the housekeeping gene EF1A as a reference to eliminate sample-to-sample variation in the cDNA samples

Consistent with the pattern in many other parasitoid species, among *A. disparis* reared indoors, females have longer lifespan than males (Fig. 4a, *Wilcoxon* = 34.646, *df* = 1, *p* < 0.001). In the wild, *A. disparis* females typically survive more than 1 month, whereas males live only for approximately 1 week [43]. The transcriptome data might provide a molecular background and suggest candidate genes for future studies of exploring the sex differences in longevity in this species. A gene encoding superoxide dismutase (SOD) (Fig. 4b, c61117.graph_c0; qRT-PCR: *t* = 2.694, *df* = 6, *p* < 0.05), which has been hypothesized to play a key role in ageing and is associated with longevity in *Drosophila* [57], was annotated and found to be upregulated in *A. disparis* females. In addition, we found that 2 genes encoding vitellogenin (Fig. 4b c67249.graph_c0: qRT-PCR, *t* = 3.955, *df* = 6, *p* < 0.05; c68140.graph_c0: qRT-PCR, *t* = 2.831, *df* = 4, *p* < 0.05), which is a yolk protein believed to play an important role in egg production, was upregulated in females [58]. In addition, *vitellogenin* plays an important role in antioxidant function related to queen longevity and is highly upregulated in the queens of diverse eusocial insect taxa [59–62].

**Fig. 5:**
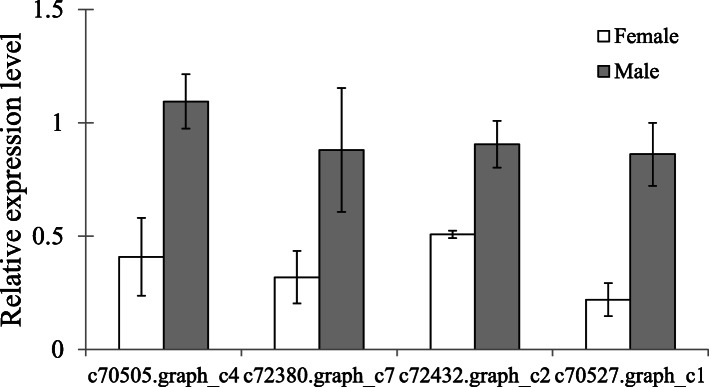
Sex differences in expression of annotated genes encoding dopamine receptor, octopamine receptor and serotonin receptor, and *muscleblind*, involving in aggression from qRT-PCR. The expression of genes determined through qRT-PCR was calculated by the 2^-ΔΔCt^ method using the housekeeping gene EF1A as a reference to eliminate sample-to-sample variations in the initial cDNA samples

Males of *A. disparis* frequently engage in extreme fighting behaviour in an attempt to acquire mating opportunities. Our transcriptome data are consistent with this observation, with the functions of some annotated genes highly expressed in males being associated with aggression, as widely studied in *D. melanogaster* [63, 64]. Male fighting in *A. disparis* is high intensity and dangerous, with contestants commonly being severely injured or killed [48, 49]. The annotated gene *muscleblind* (Fig. 5: c70505.graph_c4) was highly expressed in males and might play a role in the high-intensity male fighting in *A. disparis* (qRT-PCR: *t* = 7.244, *df* = 4, *p* < 0.05). *Muscleblind* encodes a protein with a zinc finger domain involved in muscle development; it might thus directly affect the frequency and/or intensity of aggressive interactions involving tussling or other elaborate postures [63]. Besides, a increasing number of studies have shown that biogenic amine systems, such as dopamine, octopamine, and serotonin, modulate behavioural changes in many species [65]. Our transcriptome data also showed that protein-coding genes of dopamine (c72380.graph_c7, qRT-PCR: *t* = 3.75, *df* = 4, *p* < 0.05), octopamine (c72432.graph_c2, qRT-PCR: *t* = 1.44, *df* = 4, *p* < 0.05) and serotonin (c70527.graph_c1; qRT-PCR: *t* = 4.12, *df* = 4, *p* < 0.05) receptors were unregulated in males (Fig. 5). Aggressive behaviour is widely observed in animal species; however, only a few species show dangerous fighting patterns. There have been few studies on this topic, especially those focussed on molecular mechanisms. The annotated genes in thus study could serve as candidate genes in future studies of the molecular and evolutionary mechanisms underlying dangerous fighting.

### Sex-specific genes

Among genes with sex-biased expression, some are expressed exclusively in one sex; this phenomenon has been termed sex-specific expression [6]. In most species, the male and female genomes differ by genes located on sex-specific chromosomes (such as the Y chromosome of mammals) [4, 5]. Because sex determination in hymenopteran species is haplodiploid, females and males are nearly identical genetically [6]. However, a total of 630 sex-specific genes were found in our species (Table 1), accounting for 3.98% of all of the annotated genes, with the criteria for being considered sex specific being an FPKM< 0.4 in one sex and an FPKM> 2 in the other [41]. In the present study, 518 (**Table S****3**) and 112 (**Table S****4**) were female- and male-specific genes, respectively.

Many of the male-specific genes that were functionally annotated were found to be related to venom acid phosphatases, cytochrome P450 and chitinase, suggesting functions in detoxification, defence and mate choice [41]. In addition, some annotated genes were found to be associated with reproductive functions. For example, male-specific genes encoding ejaculatory bulb-specific protein (**Table S****4****,** c52066.graph_c0) are specifically expressed in the ejaculatory bulb and seminal fluid.

GO term analysis revealed that, similar to the SB genes, many female-specific genes were enriched for reproductive functions (**Table S****5**). Increasingly, the subcategory “establishment of ommatidial planar polarity” was found to be enriched in female-specific genes involving vision function. These genes were specifically expressed in females and might be relevant to female searching for mates, food or hosts. In addition, other female-specific genes included potentially useful candidate genes for further study. For example, an annotated *toll* gene (c71387.graph_c0) was specifically expressed in females; this gene is important during embryogenesis to establish the dorsal-ventral axis and is required for innate immunity in both mammals and invertebrates [66, 67].

## Discussion

For enriching our knowledge of gender differences in *A. disparis*, sex-biased differences in gene expression were investigated using Illumina-based transcriptomic analysis. The analysis revealed that a large set of genes accounting for 50.09% of the total showed sex-biased expression (Table 1). Although *A. disparis* is a haplodiploid species with no sex chromosomes, approximately half of the genes in adults exhibit SB expression. The percentage of sex-based genes varies among species differences; for example, it is greater than 75% in *Nasonia* species and is 57% in *D. melanogaster*, which has qualitatively distinct sex chromosomes [8, 41]*.*

**Fig. 6:**
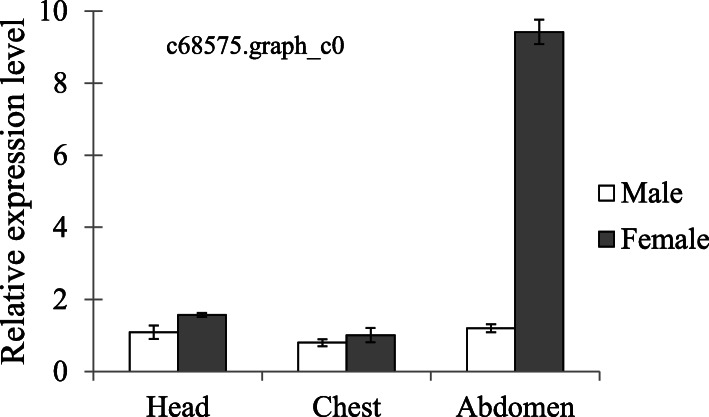
Sex differences in expression of annotated gene, Δ9-Desaturase, involving in sex pheromone production from qRT-PCR. The expression of genes determined through qRT-PCR was calculated by the 2^-ΔΔCt^ method using the housekeeping gene EF1A as a reference to eliminate sample-to-sample variations in the initial cDNA samples

Generally, the majority of sexually dimorphic traits are assumed to arise from differences in the expression of genes present in both sexes [4, 5]. Transcriptome data provided molecular background for sexually dimorphic traits such as flyability, longevity, and aggression in *A. disparis* (Figs. 3, 4, 5). Besides, previous studies [49, 68] have suggested that sex pheromones in *A. disparis* are produced by the female. Our transcriptome data also provide supporting evidence for this observation: the gene (c65173.graph_c0) encoding pheromone biosynthesis-activating neuropeptide (PBAN) exhibited extremely female-biased expression. Many studies of lepidopteran species and other insect taxa have shown that pheromone biosynthesis in females is stimulated by PBAN, a brain factor [69, 70]. In addition, many highly expressed genes encoding proteins in females in this study were related to sex-pheromone synthetic enzymes, such as acyl-CoA desaturase and short-chain dehydrogenase/reductase (**Table S****6**). One gene (c68575.graph_c0) annotated Δ9-desaturase exhibited extremely female-biased expression. Result of qRT-PCR also showed that this gene significantly highly expressed in female abdomen (Fig. 6, *t* = − 3.576, *df* = 4, *p* < 0.05). Δ9-Desaturases play important roles in the synthesis and sexual dimorphism of sex pheromones in *D. melanogaster* [71]. However, males of *Nasonia* species lack Δ9-desaturases; they have been replaced by Δ12-desaturases, which play important roles in sex pheromone production [41, 72]. Our results showed that in contrast to *Nasonia* species, *A. disparis* potentially lacks Δ12-desaturase activity, as no associated gene was annotated. Sex pheromone production in *A. disparis* females likely involves Δ9-desaturases rather than Δ12-desaturases. More studies are needed to verify these possibilities.

In addition, transcriptomic analysis revealed that accounting for 3.98% of all of the annotated genes were sex-specific genes. This percentage is similar to the range observed in *Nasonia* species of 3.8 ~ 4.8% [41]. Sex-specific genes also provided potentially useful candidate genes for further study. A prominent characteristic of parasitoids is that they can propagate on or in other arthropods. The venom of parasitoid wasps is important for the successful development of the progeny; it is injected into a host by a female wasp before or at oviposition. Many genes annotated as associated with venom proteins (see details in **Table S****7**) are related to known insect venoms and venom families in *Nasonia vitripennis* [73, 74]. Despite the large diversity of parasitoid wasp species, only a small number of venom proteins in wasps have been described. The venoms of parasitoid wasps have diverse physiological effects on their hosts, including developmental arrest, alterations in growth and physiology, suppression of immune responses, induction of paralysis, oncosis or apoptosis, and alteration of host behaviour [75–78]. An increasing number of studies have applied the venom from parasitoid wasps with the aim of developing improved biological control strategies [79]. For example, venom protein rVPr1 from the endoparasitoid *Pimpla hypochondriaca* can help control two pest insects, *Lacanobia oleracea* and *Mamestra brassicae*, in the field by suppressing the mounting of haemocyte-mediated immune responses [80, 81]. Thus, the female-specific genes annotated for venom proteins in this study may represent candidate genes for future studies of venom biology, host-parasite interactions, and biological control.

## Conclusions

In this study, we studied sex differences at the transcriptional level in an egg parasitoid species, *A. disparis*, by carrying out an Illumina-based transcriptomic analysis. The analysis revealed a large set of genes showing sex-biased expression, including a few sex-specific genes. GO enrichment analyses showed that the functional categories associated with sex-biased genes were mainly related to reproduction. The majority of sexually dimorphic traits are assumed to arise from differences in the expression of genes present in both sexes [4, 5]. This study provides comprehensive insight into the sexually dimorphic traits of parasitoid wasps (e.g., sex pheromone production, flyability, longevity, aggression) at the transcriptome level and improves our understanding of those biological traits. In addition, it provides a large number of candidate genes (e.g., *trehalase*, *muscleblind*) for future research into the molecular mechanisms underlying biological traits. Furthermore, the transcriptomic results (e.g., venom proteins) suggest the opportunity for the improved application of parasitoids to the biological control of pest species.

## Methods

### Insect materials

*Anastatus disparis* colonies were first established from a population reared on *Lymantria dispar* egg masses collected in the wild and subsequently maintained on *Antheraea pernyi* eggs [43, 44]. Most *A. disparis* adults emerge daily in the morning, primarily from 9:00 a.m. to 10:00 a.m., and we collected adults during this period.

### RNA extraction

We collected 15 adults of each sex for each of three replicates and snap-froze them in liquid nitrogen. TRIzol reagent (Invitrogen, USA) was used for extracting RNA from each group following the manufacturer’s protocol. Non-denaturing agarose gel electrophoresis and a Nanodrop 2000 spectrophotometer (Thermo Scientific, USA) were used to assess the quality and quantity of the isolated RNA, respectively. The A260/280 values were all above 2.0, and electrophoresis of the RNA samples demonstrated that the 28S and 18S rRNA were intact.

### Transcriptome sequencing and read assembly

A total of 3 μg of total RNA from each sample was converted into cDNA using the NEBNext® Ultra™ RNA Library Prep Kit for Illumina® (NEB, USA). Consequently, we constructed 6 cDNA libraries derived from three adult females and three adult males of *A. disparis*. To generate the raw reads, cDNA libraries were tagged with different adapters and then sequenced on the Illumina HiSeq 2000 platform by Beijing Biomarker Technologies Co., Ltd. Besides, an index was inserted into Illumina adapters so that all samples can be sequenced in a single lane. Approximately 8.6 Gb of paired-end reads were produced for each RNA-seq sample, and the library sizes of males and females were similar. Then, reads containing adapter, poly-N reads and low-quality reads were removed from the raw data by FASTX-Toolkit (http://hannonlab.cshl.edu/fastx_toolkit/), resulting in approximately 7.24 Gb of clean reads from each sample. The Q30 percentage was higher than 88.72% for each sample, indicating the high quality of the sequences. The high-quality reads from the six samples were pooled and assembled using Trinity software (v2.5.1) with the default parameters [82]. We chose the longest isoform of each gene to construct the unigene set. After the isoforms were selected, the assembled transcripts were predicted to be the unigenes produced. Bowtie2 was used to align the reads to the unigenes [83]. A total of 225,389 unigenes were produced; the N50 size of the unigenes was approximately 715 bp, and the mean unigene length was 570.38 bp (**Table S****8**).

### Gene expression and functional annotation

Using our assembled transcriptome as a reference, we identified putative genes expressed in males and females by RSEM [84] using the FPKM method. Functional annotation was performed by sequence similarity searching with an *E*-value cutoff of 10^− 5^ using Blastx against 8 public databases: Clusters of Orthologous Groups (COG), euKaryotic Orthologous Groups (KOG), egg NOG (v4.5), Protein family (Pfam), Swiss-Prot, NCBI non-redundant protein sequences (nr), KEGG Ortholog (KO) and GO.

### Differentially expressed genes (DEGs) and enrichment analyses

DEGs were identified using the DESeq2 package (v1.6.3) in R, and RSEM reads were incorporated into DESeq2 using tximport [85]. Genes with at least 2-fold expression changes and an FDR < 0.01 were considered differentially expressed. The GOseq R package [86] was used to determine the statistical enrichment of DEGs in the GO subcategories, and an adjusted Q-value < 0.05 was chosen as the significance cutoff.

### qRT-PCR

The expression levels of the DEGs identified in the transcriptomic analysis were evaluated by qRT-PCR. Following the abovementioned protocols, RNA from each sample group was extracted, and the concentration was measured. Then, the PrimeScript RT Reagent Kit (TaKaRa; Japan) was used to synthesize first-strand cDNA using 0.5 mg of total RNA as a template. The resultant cDNA was diluted to 0.1 mg/ml for subsequent qRT-PCR analysis (ABI StepOne Plus; USA) using SYBR Green Real-Time PCR Master Mix (TaKaRa; Japan). Primers for the selected genes (**Table S****9**) were designed using Primer Express 2.0 software. Each reaction mixture contained 0.4 μL of each primer (10 μmol/μL), 10 μL of 2 × SYBR Green Master Mix, and 2 μL of cDNA template, with water added to yield a final volume of 20 μL. The cycling parameters were 95 °C for 30 s followed by 40 cycles of 95 °C for 5 s and 62 °C for 34 s. To evaluate nonspecific product amplification, the reaction was followed by a melting curve protocol (65 °C to 95 °C in increments of 0.5 °C every 5 s). Relative gene expression was calculated by the 2^-ΔΔCt^ method. The housekeeping gene translation elongation factor 1-α (EF1A) was used as a reference to eliminate sample-to-sample variation in the initial cDNA samples.

### Longevity assay

For the longevity assays, males and females (1 day old) were selected at 10:00 a.m. Each male was isolated individually in a cylindrical box (height: 5 cm, diameter: 10 cm) and received honey water daily (honey: water = 2:3 vol/vol). Each male was inspected twice daily, at 10 a.m. and 10 p.m., and the date of death of each male was recorded.

### Statistical analysis

Prior to analysis, the raw data were tested for normality and homogeneity of variances using Kolmogorov-Smirnov and Levene’s tests, respectively, and the data were transformed where necessary. The qRT-PCR data of gene expression were compared between males and females with independent *t*-tests. Survival analysis was applied to analyse the sex difference in longevity. All statistical analyses were performed using SPSS software (version 20).

## Supplementary information

**Additional file 1: Table S1.** Annotated female biased genes in *Anastatus disparis*.

**Additional file 2: Table S2.** Annotated male biased genes in *Anastatus disparis*.

**Additional file 3: Table S3.** Annotated female-specific genes in *Anastatus disparis*.

**Additional file 4: Table S4.** Annotated male specific-genes in *Anastatus disparis*.

**Additional file 5: Table S5.** Annotated female-specific genes in enrich GO subcategories.

**Additional file 6: Table S6.** Annotated sex biased genes related to sex-pheromone synthetic enzymes.

**Additional file 7: Table S7.** Annotated female-specific genes involving encoding venom proteins.

**Additional file 8: Table S8.** Statistics of transcriptome assembly and predicted unigenes.

**Additional file 9: Table S9.** Primer pairs used for expression analysis using qRT-PCR.

## Data Availability

The data sets used and/or analysed during the current study are available from the corresponding author on reasonable request. All raw sequence data files are available from the Sequence Read Archive database (BioProject ID PRJNA642922; https://www.ncbi.nlm.nih.gov/sra/PRJNA642922).

## Abbreviations

GO: Gene Ontology
MF: Molecular Function
CC: Cell Component
BP: Biological Process
FDR: False discovery rate
DEGs: Differentially expressed genes
qRT-PCR: quantitative real time polymerase chain reaction
EF1A: Translation elongation factor 1-α
FPKM: Fragments Per Kilobase of transcript per Million mapped reads
SB: Sex-biased
PBAN: Pheromone biosynthesis-activating neuropeptide
SOD: Superoxide dismutase
COG: Clusters of Orthologous Groups,
KOG: euKaryotic Orthologous Groups
Pfam: Protein family
NR: NCBI non-redundant protein sequences
KO: KEGG Ortholog

## References

[1] Breedlove SM (1992). Sexual dimorphism in the vertebrate nervous-system. J Neurosci.

[2] Bardin CW, Catterall JF (1981). Testosterone: a major determinant of extragenital sexual dimorphism. Science.

[3] Darwin CR (1871). The descent of man, and selection in relation to sex (Murray, London).

[4] Connallon T, Knowles LL (2005). Intergenomic conflict revealed by patterns of sex-biased gene expression. Trends Genet.

[5] Rinn JL, Snyder M (2005). Sexual dimorphism in mammalian gene expression. Trends Genet.

[6] Ellegren H, Parsch J (2007). The evolution of sex-biased genes and sex-biased gene expression. Nat Rev Genet.

[7] Arbeitman M, Fleming A, Siegal M, Null B, Baker B (2004). A genomic analysis of Drosophila somatic sexual differentiation and its regulation. Development..

[8] Ranz J, Castillo-Davis C, Meiklejohn C, Hartl D (2003). Sex-dependent gene expression and evolution of the *Drosophila* transcriptome. Science..

[9] Hahn MW, Lanzaro GC (2005). Female-biased gene expression in the malaria mosquito *Anopheles gambiae*. Curr Biol.

[10] Eads BD, Colbourne JK, Bohuski E, Andrews J (2007). Profiling sex-biased gene expression during parthenogenetic reproduction in *Daphnia pulex*. BMC Genomics.

[11] Parisi M, Nuttall R, Edwards P, Minor J, Naiman D, Lü J (2004). A survey of ovary-, testis-, and soma-biased gene expression in *Drosophila melanogaster* adults. Genome Boil.

[12] Allen SL, Bonduriansky R, Chenoweth SF (2018). Genetic constraints on microevolutionary divergence of sex-biased gene expression. Philos T R Soc B.

[13] Baker DA, Nolan T, Fischer B, Pinder A, Crisanti A, Russell S (2011). A comprehensive gene expression atlas of sex- and tissue-specificity in the malaria vector, *Anopheles gambiae*. BMC Genomics.

[14] Prince EG, Kirkland D, Demuth JP (2010). Hyperexpression of the X chromosome in both sexes results in extensive female bias of X-linked genes in the flour beetle. Genome Biol Evol.

[15] Hambuch T, Parsch J (2005). Patterns of synonymous codon usage in *Drosophila melanogaster* genes with sex-biased expression. Genetics.

[16] Zhang Z, Hambuch T, Parsch J (2005). Molecular evolution of sex-biased genes in *Drosophila*. J Mol Evol.

[17] Meiklejohn CD, Parsch J, Ranz JM, Hartl DL (2003). Rapid evolution of male-biased gene expression in *Drosophila*. P Natl Acad Sci USA.

[18] Davis J, Brandman O, Petrov D (2005). Protein evolution in the context of *Drosophila* development. J Mol Evol.

[19] Allen SL, McGuigan K, Connallon T, Blows MW, Chenoweth SF (2017). Sexual selection on spontaneous mutations strengthens the between-sex genetic correlation for fitness. Evolution.

[20] Lipinska A, Cormier A, Luthringer R, Peters AF, Corre E, Gachon CM, Cock JM, Coelho SM (2015). Sexual dimorphism and the evolution of sex-biased gene expression in the brown alga ectocarpus. Mol Biol Evol.

[21] Pointer MA, Harrison PW, Wright AE, Mank JE (2013). Masculinization of gene expression is associated with exaggeration of male sexual dimorphism. PLoS Genet.

[22] Uebbing S, Kunstner A, Makinen H, Ellegren H (2013). Transcriptome sequencing reveals the character of incomplete dosage compensation across multiple tissues in flycatchers. Genome Biol Evol.

[23] Albritton SE, Kranz AL, Rao P, Kramer M, Dieterich C, Ercan S (2014). Sex-biased gene expression and evolution of the x chromosome in nematodes. Genetics..

[24] Bohne A, Sengstag T, Salzburger W (2014). Comparative transcriptomics in east African cichlids reveals sex- and species-specific expression and new candidates for sex differentiation in fishes. Genome Biol Evol.

[25] Sharma E, Kunstner A, Fraser BA, Zipprich G, Kottler VA, Henz SR, Weigel D, Dreyer C (2014). Transcriptome assemblies for studying sex-biased gene expression in the guppy, *Poecilia reticulata*. BMC Genomics.

[26] Smith G, Chen YR, Blissard GW, Briscoe AD (2014). Complete dosage compensation and sex-biased gene expression in the moth *Manduca sexta*. Genome Biol Evol.

[27] Jaquiery J, Rispe C, Roze D, Legeai F (2013). Masculinization of the x chromosome in the pea aphid. PLoS Genet.

[28] Jin W, Riley RM, Wolfinger RD, White KP, Passadorgurgel G, Gibson G (2001). The contributions of sex, genotype and age to transcriptional variance in *Drosophila melanogaster*. Nat Genet.

[29] Arbeitman MN, Furlong EE, Imam F, Johnson E, Null BH, Baker BS, Krasnow MA, Scott MP, Davis RW, White KP (2002). Gene expression during the life cycle of *Drosophila melanogaster*. Science..

[30] Chang PL, Dunham JP, Nuzhdin SV, Arbeitman MN (2011). Somatic sex specific transcriptome differences in *Drosophila* revealed by whole transcriptome sequencing. BMC Genomics.

[31] Perry JC, Harrison PW, Mank JE (2014). The ontogeny and evolution of sex-biased gene expression in *Drosophila melanogaster*. Mol Biol Evol.

[32] Wen X, Guo L, Jiao X, Yang N, Xin Y, Wu Q, Wang S, Zhou X, Zhang Y (2014). Transcriptomic dissection of sexual differences in *Bemisia tabaci*, an invasive agricultural pest worldwide. Sci Rep.

[33] Liu PC, Tian S, Hao D (2019). Sexual transcription differences in *Brachymeria lasus* (Hymenoptera: Chalcididae), a pupal parasitoid species of *Lymantria dispar* (Lepidoptera: Lymantriidae). Front Genet.

[34] Godfray HCJ (1994). Parasitoids: Behavioural and evolutionary ecology.

[35] Terayama M, Seiki Y, Ikudome S, Terayama M (1999). Description of new species of the family Bethylidae from the Ryukyus, and taxonomic notes on the Japanese species of the genus *Sclerodermus*. Identification guide to the Aculeata of the Nansei Islands, Japan.

[36] Hassan SA (1993). The mass rearing and utilization of *Trichogramma* to control lepidopterous pests: achievements and outlook. Pest Manag Sci.

[37] Lim JO, Lyu DP, Choi GS, Jeong YJ, Shin SC, Lee SH (2006). A taxonomie note on *Sclerodermas harmandi*, ectoparasite of stem and wood boring insect larvae (Hymenoptera: Chrysidoidea’-Bethylidae) in South Korea. J Asia Pac Entomol.

[38] Li L, Wajnberg E, Hassan SA (1994). Worldwide use of *Trichogramma* for biological control on different crops: a survey. Biological control with egg parasitoids.

[39] Zhishan W, Hopper KR, Ode PJ, Fuester RW, Jia-Hua C, Heimpel GE (2003). Complementary sex determination in hymenopteran parasitoids and its implications for biological control. Entomol Sin.

[40] Parra JRP, Zucchi AR (2004). *Trichogramma* in Brazil: feasibility of use after twenty years of research. Neotrop Entomol.

[41] Wang X, Werren JH, Clark AG (2015). Genetic and epigenetic architecture of sex-biased expression in the jewel wasps *Nasonia vitripennis* and *giraulti*. P Natl Acad Sci USA.

[42] Cook JM (1993). Sex determination in the Hymenoptera: a review of models and evidence. Heredity..

[43] Yan JJ, Xu CH, Gao WC, Li GW, Yao DF, Zhang PY (1989). Parasites and predators of forest pest.

[44] Li BJ, Lou JX. Preliminary studies on *Anastatus disparis* (Hymenoptera: Eupelmidae), an egg parasitoid of gypsy moth*. Chin*. J Biol Cont. 1992;144.

[45] Crossman SS (1925). Two imported egg parasites of the gipsy moth, *Anastatus bifasciatus* Fonsc and *Schedius kuvanae* Howard. J Agr Res.

[46] Liu PC, Men J, Zhao B, Wei JR (2017). Fitness-related offspring sex allocation of *Anastatus disparis*, a gypsy moth egg parasitoid, on different-sized host species. Entomol Exp Appl.

[47] Liu PC, Wei JR, Wang JJ, Liu JX, Dong LJ (2015). Relationship between the environmental temperatures and development of *Anastatus disparis* (Ruschka) (Hymenoptera: Eupelmidae) and the sex ratio control of the offspring. Forest Pest Dis.

[48] Liu PC, Wei JR, Tian S, Hao DJ (2017). Male-male lethal combat in the quasi-gregarious parasitoid *Anastatus disparis* (Hymenoptera: Eupelmidae). Sci Rep.

[49] Liu PC, Hao DJ (2019). Effect of variation in objective resource value on extreme male combat in a quasi-gregarious species, *Anastatus disparis*. BMC Ecology.

[50] Bréque C, Surai P, Brillard JP (2003). Roles of antioxidants on prolonged storage of avian spermatozoa in vivo and in vitro. Mol Reprod Dev.

[51] Chen H, Cheung MP, Chow PH, Cheung AL, Liu W (2002). Protection of sperm DNA against oxidative stress in vivo by accessory sex gland secretions in male hamsters. Reproduction.

[52] Spradling AC. Developmental genetics of oogenesis. In: Bate M, Martinez-Arias A, editors. The Development of Drosophila. New York: Cold Spring Harbor Laboratory Press; 1993. p. 1–70.

[53] Saboe-Larssen S, Lyamouri M, Merriam J, Oksvold MP, Lambertsson A (1998). Ribosomal protein insufficiency and the minute syndrome in *Drosophila*: a dose-response relationship. Genetics.

[54] Brook WJ, Diaz-Benjumea FJ, Cohen SM (1996). Organizing spatial pattern in limb development. Annu Rev Cell Dev Biol.

[55] Brisson JA, Davis GK, Stern DL (2007). Common genome-wide patterns of transcript accumulation underlying the wing polyphenism and polymorphism in the pea aphid (Acyrthosiphon pisum). Evol Dev.

[56] Yang X, Liu X, Xu X (2014). Gene expression profiling in winged and wingless cotton aphids, aphis gossypii (Hemiptera: Aphididae). Int J Biol Sci.

[57] Orr WC, Sohal RS (1994). Extension of life-span by overexpression of superoxide dismutase and catalase in *Drosophila melanogaster*. Science.

[58] Foster KR, Ratnieks FLW, Gyllenstrand N, Thoren PA (2001). Colony kin structure and male production in *Dolichovespula wasps*. Mol Ecol.

[59] Scharf ME, Wu-Scharf D, Zhou X, Pittendrigh BR, Bennett GW (2005). Gene expression profiles among immature and adult reproductive castes of the termite *Reticulitermes flavipes*. Insect Mol Biol.

[60] Sumner S, Pereboom JJM, Jordan WC (2006). Differential gene expression and phenotypic plasticity in behavioural castes of the primitively eusocial wasp, Polistes canadensis. Proc R Soc B.

[61] Corona M, Velarde RA, Remolina S, Moran-Lauter A, Wang Y, Hughes KA (2007). Vitellogenin, juvenile hormone, insulin signaling, and queen honey bee longevity. Proc Natl Acad Sci U S A.

[62] Graff J, Jemielity S, Parker JD, Parker KM, Keller L (2007). Differential gene expression between adult queens and workers in the ant *Lasius niger*. Mol Ecol.

[63] Edwards AC, Rollmann SM, Morgan TJ, Mackay TF. Quantitative genomics of aggressive behavior in *Drosophila melanogaster*. PLoS Genet. 2006;2(9):1386-95. 10.1371/journal.pgen.0020154 PMC1564424 17044737

[64] Edwards AC, Zwarts L, Yamamoto A, Callaerts P, Mackay TF (2009). Mutations in many genes affect aggressive behavior in *Drosophila melanogaster*. BMC Biol.

[65] Kim YK (2016). A *Drosophila* Model for Aggression. Animal Models of Behavior Genetics.

[66] Lemaitre B, Nicolas E, Michaut L, Reichhart JM, Hoffmann JA (1996). The dorsoventral regulatory gene cassette spätzle/toll/cactus controls the potent antifungal response in *Drosophila* adults. Cell..

[67] Medzhitov R, Preston-Hurlburt P, Janeway CA (1997). A human homologue of the *Drosophila* toll protein signals activation of adaptive immunity. Nature.

[68] Liu PC, Hao DJ (2019). Behavioural and transcriptional changes in post-mating females of an egg parasitoid wasp species. Roy Soc Open Sci.

[69] Rafaeli A (2009). Pheromone biosynthesis activating neuropeptide (PBAN): regulatory role and mode of action. Gen Comp Endocr.

[70] Choi MY, Vander Meer RK (2012). Molecular structure and diversity of PBAN/pyrokinin family peptides in ants. Front Endocrinol.

[71] Labeur C, Dallerac R, Wicker-Thomas C (2002). Involvement of desat1 gene in the control of *Drosophila melanogaster* pheromone biosynthesis. Genetica..

[72] Blaul B, Steinbauer R, Merkl P, Merkl R, Tschochner H, Ruther J (2014). Oleic acid is a precursor of linoleic acid and the male sex pheromone in *Nasonia vitripennis*. Insect Biochem Molec.

[73] Graaf DCD, Aerts M, Brunain M, Desjardins CA, Jacobs FJ, Werren JH, Devreese B (2010). Insights into the venom composition of the ectoparasitoid wasp *Nasonia vitripennis* from bioinformatic and proteomic studies. (special issue: the *Nasonia* genome.). Insect Mol Biol.

[74] Werren JH, Richards S, Desjardins CA (2010). Functional and evolutionary insights from the genomes of three parasitoid *Nasonia* species. Science..

[75] Tian C, Wang L, Ye G, Zhu S (2010). Inhibition of melanization by a *Nasonia* defensin-like peptide: implications for host immune suppression. J Insect Physiol.

[76] Kryukova N, Dubovskiy I, Chertkova E, Vorontsova Y, Slepneva I, Glupov V (2011). The effect of *Habrobracon hebetor* venom on the activity of the prophenoloxidase system, the generation of reactive oxygen species and encapsulation in the haemolymph of *Galleria mellonella* larvae. J Insect Physiol.

[77] Edwards JP, Bell HA, Audsley N, Marris GC, Kirkbride-Smith A, Bryning G, Frisco C, Cusson M (2006). The ectoparasitic wasp *Eldophus pennicornis* (Hymenoptera: Eulophiclae) uses instar-specific endocrine disruption strategies to suppress the development of its host *Lacanobia oleracea* (Lepidoptera: Noctuidae). J Insect Physiol.

[78] Price D, Bell H, Hinchliffe G, Fitches E, Weaver R, Gatehouse JA (2009). Venom metalloproteinase from the parasitic wasp *Eulophus pennicornis* is toxic towards its host, tomato moth (*Lacanobia oleracae*). Insect Mol Biol.

[79] Moreau SJM, Asgari S (2015). Venom proteins from parasitoid wasps and their biological functions. Toxins.

[80] Richards EH, Bradish H, Dani MP, Pietravalle S, Lawson A (2011). Recombinant immunosuppressive protein from *Pimpla hypochondrica* venom (rVPr1) increases the susceptibility of *Mamestra brassicae* larvae to the fungal biological control agent. Beauveria bassiana Arch Insect Biochem.

[81] Richards EH, Dani MP, Bradish H (2013). Immunosuppressive properties of a protein (rVPr1) from the venom of the endoparasitic wasp, *Pimpla hypochondriaca*: mechanism of action and potential use for improving biological control strategies. J Insect Physiol.

[82] Grabherr MG, Haas BJ, Yassour M, Levin JZ, Thompson DA, Amit I (2011). Full-length transcriptome assembly from RNA-seq data without a reference genome. Nat Biotechnol.

[83] Langmead B, Trapnell C, Pop M, Salzberg SL (2009). Ultrafast and memory-efficient alignment of short DNA sequences to the human genome. Genome Boil.

[84] Li B, Dewey CN (2011). RSEM: accurate transcript quantification from RNA-Seq data with or without a reference genome. BMC Bioinformatics.

[85] Soneson C, Love MI, Robinson MD. Differential analyses for RNA-seq: transcript-level estimates improve gene-level inferences. F1000Res. 2015;4:1521. 10.12688/f1000research.7563.1 PMC4712774 26925227

[86] Young MD, Wakefield MJ, Smyth GK, Oshlack A (2010). Gene ontology analysis for rna-seq: accounting for selection bias. Genome Biol.

