# Supplementary material for: Sexual dimorphism and sex-biased gene expression in an egg parasitoid species, Anastatus disparis
Source: BMC Genomics. 2020 Jul 18;21:492. doi: 10.1186/s12864-020-06903-5 (PMC7368684; doi:10.1186/s12864-020-06903-5)
Supplement: Supplementary file 8 — Additional file 8: Table S8. Statistics of transcriptome assembly and predicted unigenes. [file 12864_2020_6903_MOESM8_ESM.docx]

**Table S8.** Statistics of transcriptome assembly and predicted unigenes

| Length Range | Transcripts | Unigenes |
| --- | --- | --- |
| 200-300 bp | 107,627(31.80%) | 96,604(42.86%) |
| 300-500 bp | 82,020(24.24%) | 69,412(30.80%) |
| 500-1000 bp | 55,088(16.28%) | 37,422(16.60%) |
| 1000-2000 bp | 33,674(9.95%) | 13,172(5.84%) |
| >2000 bp | 59,991(17.73%) | 8,779(3.90%) |
| Total Number | 338,400 | 225,389 |
| Size of data (bp) | 425,770,709 | 128,558,149 |
| N50 length (bp) | 3,533 | 715 |
| Mean length (bp) | 1258.19 | 570.38 |
