# Supplementary material for: Sexual dimorphism and sex-biased gene expression in an egg parasitoid species, Anastatus disparis
Source: BMC Genomics. 2020 Jul 18;21:492. doi: 10.1186/s12864-020-06903-5 (PMC7368684; doi:10.1186/s12864-020-06903-5)
Supplement: Supplementary file 9 — Additional file 9: Table S9. Primer pairs used for expression analysis using qRT-PCR. [file 12864_2020_6903_MOESM9_ESM.docx]

**Table S9.** Primer pairs used for expression analysis using qRT-PCR

| Gene name | Primer sequences |
| --- | --- |
| c42845.graph_c0 | Forward: 5’- CGACCTGTCGTGACACTTTC-3’ |
|  | Reverse: 5’- ATGAGCACTTGCTGAAGCTG-3’ |
| c72605.graph_c2 | Reverse: 5’- TAGCAAGACCAACGTCACCT-3’ |
|  | Forward: 5’- CCGTTAGTCCAGCCAAATCC-3’ |
| c23296.graph_c0 | Forward: 5’- CCGTTAGTCCAGCCAAATCC-3’ |
|  | Reverse: 5’- AGAGGCTTGCTACTCTGTGG-3’ |
| c66701.graph_c0 | Forward: 5’- TGATGTGTTTCACAAACTGCAA-3’ |
|  | Reverse: 5’- TGACATTGAGGCTTGGATGC-3’ |
| c61117.graph_c0 | Forward: 5’-GGGCTCGAGTGCCTTATAGT -3’ |
|  | Reverse: 5’- CTGCCAAGCGTGCTATTGTC-3’ |
| c67249.graph_c0 | Forward: 5’- ATTCCGTGATGCTGTTGCTC-3’ |
|  | Reverse: 5’-AACTTGGGTGGAATCCGCTA -3’ |
| c68140.graph_c0 | Forward: 5’- ATGAACGCGAACGTAAGCAA -3’ |
|  | Reverse: 5’- TCAACGTCGCTCATTCCAAC -3’ |
| c68575.graph_c0 | Forward: 5’- GTGCGCAAGAGTCCAGTCGT-3’ |
|  | Reverse: 5’- GGTGCTCATCGACTCTGGGC-3’ |
| c70505.graph_c4 | Forward: 5’- TCCAACGGGTGTTGGCAGTC-3’ |
|  | Reverse: 5’- CGCCACCTGACGCATGTAGT-3’ |
| c72380.graph_c7 | Forward: 5’- TGACGGTCTGGATGCTTGCC-3’ |
|  | Reverse: 5’- AATTGCGTACGTGGGCGTCA-3’ |
| c72432.graph_c2 | Forward: 5’- CAAAAACGCCGTTCCACGCA-3’ |
|  | Reverse: 5’- GTACGCGCTGCTTTGTGCTC-3’ |
| c70527.graph_c1 | Forward: 5’- TTCAACCACCACGCGAACGA-3’ |
|  | Reverse: 5’- ATGGTGGTGCCGGATTAGCG-3’ |
| EF1A | Forward: 5’- ACCACGAAGCTCTCCAAGAA-3’ |
|  | Reverse: 5’- AATCTGCAGCACCCTTAGGT-3’ |
